# Gut-brain-liver axis in growth hormone deficiency: role of microbiota-derived short-chain fatty acids in ethnic variability and therapeutic development

**DOI:** 10.3389/fpubh.2025.1541654

**Published:** 2025-05-30

**Authors:** Dongming Meng, Wei Kong, Sai Cheng, Hua Liu, Congfu Huang

**Affiliations:** Department of Pediatrics, Longgang Maternity and Child Institute of Shantou University Medical College (Longgang District Maternity & Child Healthcare Hospital of Shenzhen City), Shenzhen, China

**Keywords:** growth hormone deficiency, gut microbiota, short-chain fatty acids, gut-brain axis, insulin-like growth factor-1, therapeutic interventions

## Abstract

Growth hormone deficiency (GHD) is a pediatric endocrine disorder characterized by dysregulated growth hormone/insulin-like growth factor-1 (GH/IGF-1) axis activity and gut microbiota imbalance. Emerging evidence highlights the gut-brain-liver axis as a critical modulator of growth, with microbiota-derived short-chain fatty acids (SCFAs) playing dual roles in GH suppression and IGF-1 enhancement. This review synthesizes preclinical and clinical data to address ethnic variability in microbiota composition and therapeutic challenges. Key findings reveal that Chinese GHD cohorts exhibit reduced *Bifidobacterium* and fecal butyrate, whereas Spanish cohorts show minimal differences, potentially due to dietary fiber intake (e.g., *Prevotella*-enriched diets in Asia) or methodological variations in microbiota sequencing. Mechanistically, propionate (>500 μM) inhibits pituitary GH synthesis via GPR41/43-cAMP signaling, while butyrate enhances hepatic IGF-1 through GPR109A-mediated IL-6 secretion and osteoblastic histone deacetylase (HDAC) inhibition. Interventions such as probiotics (e.g., *Lactobacillus plantarum* increased IGF-1 by 1.2–1.8-fold in murine models) and high-fiber diets demonstrate preclinical efficacy but face clinical barriers, including poor adherence (<30%) and limited GHD-specific trials. Fecal microbiota transplantation (FMT) shows hormonal restoration in animal models but induces gastrointestinal adverse effects (22% bloating, 15% diarrhea) in humans. Multi-omics approaches are proposed to identify biomarkers (e.g., low butyrate + elevated trimethylamine N-oxide). These approaches also aim to optimize precision therapies, such as nanoparticle-delivered SCFAs. This review underscores the need for multicenter randomized controlled trials to validate synbiotics or engineered microbial consortia, bridging mechanistic insights into the microbiota-SCFA-endocrine axis with clinical translation for GHD management.

## 1 Introduction

Growth hormone deficiency (GHD) is a pediatric endocrine disorder characterized by insufficient secretion of growth hormone (GH), which results in impaired linear growth, metabolic dysregulation, and a reduced quality of life (1, 2). The hypothalamus-GH-IGF-1 axis is central to understanding the pathogenesis of GHD. However, emerging evidence highlights the gut microbiota as a crucial modulator of growth through its metabolic and endocrine functions (3, 4).

The gut microbiota produces a variety of metabolites, including short-chain fatty acids (SCFAs), which influence host physiology by regulating nutrient absorption, immune responses, and hormonal signaling (5, 6). Specifically, among the SCFAs—acetate, propionate, and butyrate—propionate suppresses pituitary GH synthesis via GPR41/43-cAMP pathways (7), while butyrate enhances hepatic IGF-1 production through epigenetic modulation (8).

Clinical studies, however, have reported contradictory findings. For example, Huang et al. (9) observed reduced levels of *Bifidobacterium* (a key producer of SCFAs) and fecal butyrate in Chinese children with GHD. In contrast, García Navas et al. (10) found no significant alterations in the gut microbiota of Spanish cohorts, suggesting potential ethnic or methodological variability.

Beyond SCFAs, other microbial metabolites, such as secondary bile acids and tryptophan derivatives (e.g., indole-3-propionic acid), also modulate growth pathways. Indole-3-propionic acid, a tryptophan derivative, mitigates TNF-*α*-mediated GH suppression by activating the aryl hydrocarbon receptor (AhR), thereby reducing systemic inflammation and supporting growth hormone signaling. Secondary bile acids inhibit hepatic GH receptor activity via FXR signaling (11), while indole derivatives mitigate TNF-*α*-mediated GH suppression through the activation of aryl hydrocarbon receptor (AhR) (12, 13). Current research predominantly focuses on SCFAs, leaving gaps in our understanding of the synergistic effects of other metabolites. Specific gut bacterial species, such as *Bifidobacterium* and *Lactobacillus*, have been identified as key players in modulating GH/IGF-1 axis activity through their production of SCFAs. Dysbiosis of these species is frequently observed in pediatric GHD cohorts.

This review systematically synthesizes the role of the “microbiota-SCFA-endocrine” axis in growth hormone deficiency (GHD), addressing three critical research gaps:

1. **Mechanistic Complexity**: The bidirectional interactions between microbial metabolites and hormonal pathways, particularly the interplay between short-chain fatty acids (SCFAs) and the hypothalamus-GH-IGF-1 axis, remain incompletely understood. Further investigation is needed to elucidate how microbial metabolites influence endocrine signaling and vice versa.
2. **Clinical Contradictions**: Discrepancies in clinical findings highlight the impact of ethnic and dietary variability on gut microbiota composition. These factors may explain inconsistencies in microbiota profiles observed across different populations and underscore the need for standardized methodologies in future studies.
3. **Therapeutic Challenges**: The translation of probiotics, dietary fiber, and fecal microbiota transplantation (FMT) into effective therapeutic strategies for GHD is limited by gaps in understanding microbial dysbiosis and its functional consequences. Addressing these challenges requires a more nuanced approach to harnessing microbial interventions.

By integrating preclinical and clinical evidence, this review emphasizes the necessity of multi-omics approaches to identify GHD-specific biomarkers and develop precision therapies targeting microbial dysbiosis (an imbalance in gut microbiota composition and function). We prioritize SCFAs due to their well-documented, receptor-mediated effects on pituitary GH synthesis (via GPR41/43) and hepatic IGF-1 production (via GPR109A), as supported by dose–response studies (7). However, the roles of polyamines and branched-chain fatty acids in GHD remain underexplored and warrant further investigation.

## 2 Methods

### 2.1 Literature search and analysis

A systematic literature review was conducted using PubMed, CNKI, and Wanfang databases from January 2010 to October 2024. The search strategy employed Boolean operators with the following terms:

1. Primary search terms: (“growth hormone deficiency” OR “GHD”) AND (“gut microbiota” OR “short-chain fatty acids” OR “SCFAs”) OR (“GHD AND microbiome NOT obesity”).
2. Secondary search filter: (“insulin-like growth factor-1” OR “IGF-1”) AND (“probiotics” OR “dietary fiber” OR “fecal microbiota transplantation”).

### 2.2 Inclusion and exclusion criteria

#### 2.2.1 Inclusion criteria

Studies were included if they:(1) Focused on pediatric populations or animal models of GHD; (2) Provided mechanistic insights into gut microbiota-SCFA-GH/IGF-1 interactions; and (3) Were published in English or Chinese.

#### 2.2.2 Exclusion criteria

Studies were excluded if they: (1) Were non-peer-reviewed articles, conference abstracts, or editorials; (2) Lacked quantitative data on SCFA levels or hormonal outcomes; and (3) Included animal studies constituting more than 30% of the total analyzed literature to prioritize human relevance.

### 2.3 Data synthesis

A thematic analysis was conducted to organize findings into three key domains:

1. **Microbial Dysbiosis in GHD**: Discrepancies in gut microbiota composition across human cohorts [e.g., Huang et al. (9) vs. García Navas et al. (10)] were systematically compared and tabulated to highlight potential ethnic differences and methodological inconsistencies in study design.
2. **SCFA-Mediated Mechanisms**: The dose–response relationships between SCFAs and the GH/IGF-1 axis were analyzed through integration of *in vitro* and *in vivo* experimental data (6, 14).
3. **Intervention Efficacy**: Outcomes of probiotic and dietary fiber interventions were stratified based on study design (randomized controlled trials vs. observational studies) and population characteristics (GHD vs. non-GHD cohorts) (15, 16).

## 3 Mechanism of gut microbiota affecting growth and development

### 3.1 Gut microbiota is involved in nutrition absorption and metabolism

**Figure 1:**
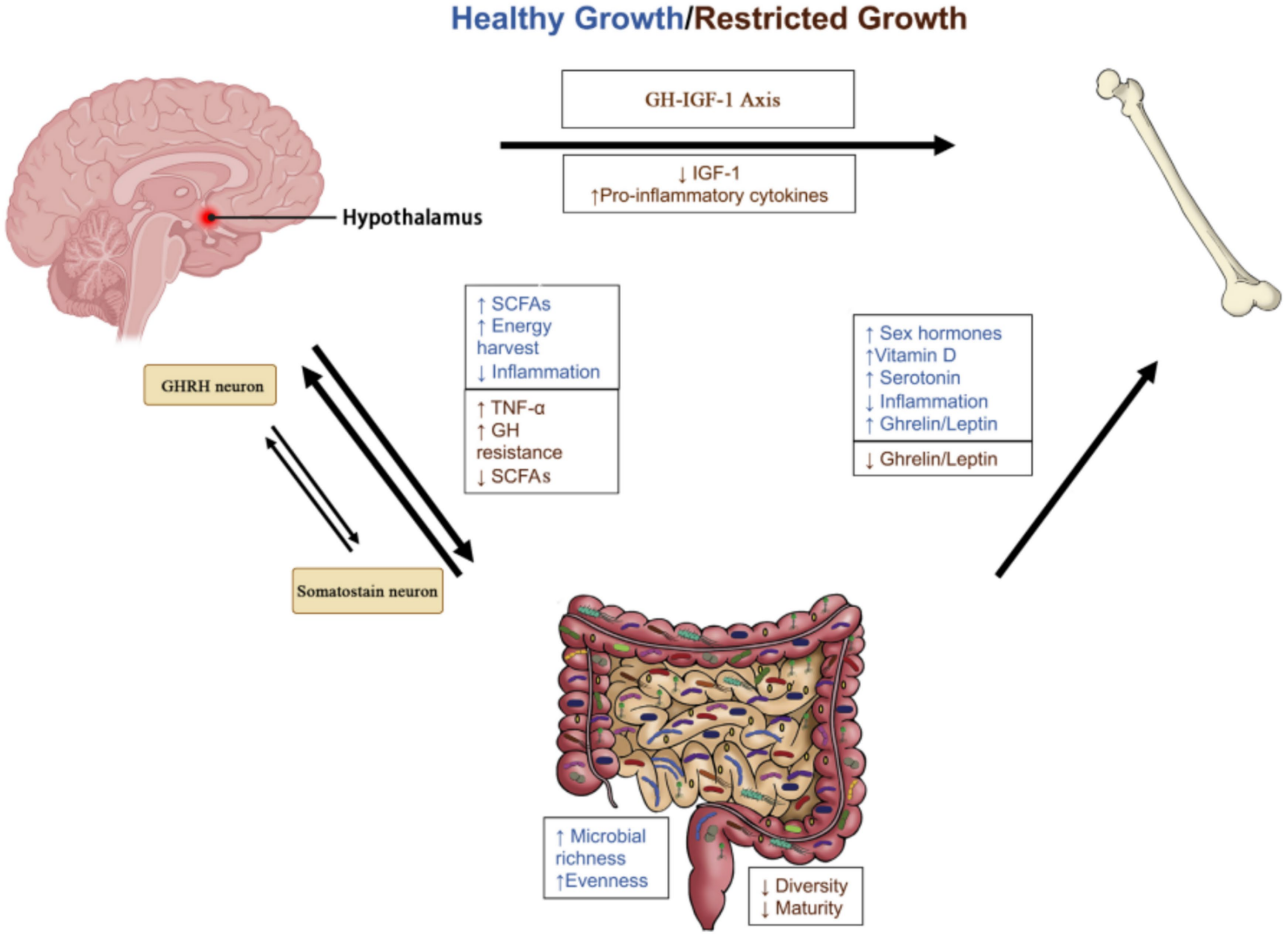
The gut microbiota modulates the hypothalamic GHRH/somatostatin balance via SCFA-GPR41/43-cAMP signaling and enhances hepatic IGF-1 through butyrate-GPR109A-IL-6 pathways. Key pathways: GH suppression via cAMP/PKA/CREB; IGF-1 upregulation via HDAC inhibition. IGF-1, insulin-like growth factor-1; SCFAs, short-chain fatty acids; TNF-α, tumor necrosis factor-α.

The gut microbiota is crucial for nutrient absorption and metabolism, directly affecting host growth and development. Healthy gut microbiota promotes linear growth and weight gain through three interconnected mechanisms (3) (Figure 1):

1. **Nutrient Competition and Appetite Regulation**: Gut microbiota harness dietary nutrients (e.g., polysaccharides, proteins) for their proliferation. In return, their metabolic demands modulate host appetite through ghrelin and peptide YY signaling (17, 18).
2. **Vitamin and Mineral Bioavailability**: Probiotics synthesize essential vitamins (A, B, C, D, E, K) vital for calcium metabolism and bone formation (19). Additionally, phytase enzymes produced by *Bifidobacterium* and *Lactobacillus* break down phytic acid, releasing minerals (e.g., phosphorus) and enhancing absorption efficiency (19).
3. **SCFA Synthesis**: *Bacteroides* and *Firmicutes* ferment undigested carbohydrates and proteins to produce short-chain fatty acids (acetate, propionate, butyrate). These SCFAs serve as energy sources for colonocytes, regulate intestinal pH, and maintain mucosal integrity (4, 5). Moreover, SCFAs influence endocrine functions by stimulating Glucagon-Like Peptide-1 (GLP-1) secretion and inhibiting pro-inflammatory cytokines (e.g., TNF-*α*, IL-6) (6, 20).

### 3.2 Bidirectional interaction between gut microbiota and hormones

The gut microbiota functions as a dynamic endocrine organ, participating in bidirectional communication with the host’s hormonal system. Microbial metabolites interact directly with enteroendocrine cells, influencing the secretion of hormones related to growth:

1. **Ghrelin Regulation**: Research indicates that germ-free mice have notably lower plasma ghrelin levels compared to conventionally raised mice, underscoring the microbiota’s role in ghrelin production (11). Ghrelin, a peptide hormone primarily secreted by the stomach, stimulates growth hormone release and regulates appetite. Among the microbial metabolites, short-chain fatty acids (SCFAs), especially propionate, suppress vagal nerve-mediated ghrelin secretion, consequently decreasing growth hormone (GH) release during fasting (21, 22).
2. **GH-IGF-1 Axis Modulation**: SCFAs, when absorbed through the portal circulation, stimulate hepatic IGF-1 biosynthesis. In children aged 6–9 years, there is a positive correlation between fecal SCFA levels and serum IGF-1 concentrations (12, 23).
3. **Feedback Loop**: A deficiency in growth hormone can modify the composition of the gut microbiota. For instance, male GH-deficient mice display a marked reduction in *Lactobacillus* spp. (e.g., *Lactobacillus rhamnosus, Lactobacillus plantarum*) abundance and an increase in Clostridiaceae family members (e.g., *Clostridium difficile*), changes that are reversible with GH therapy (24, 25). On the other hand, IGF-1 knockout models exhibit dysbiosis, characterized by reduced *Bacteroidetes* (e.g., *Bacteroides fragilis*) to *Firmicutes* (e.g., *Clostridium* spp.) ratios and compromised barrier function (26). These specific bacterial shifts are critical as *Lactobacillus* species are key producers of SCFAs and exert anti-inflammatory effects, while increased *Clostridiaceae* may promote gut barrier dysfunction and systemic inflammation, further exacerbating GH deficiency. While human clinical data on GH-microbiota feedback loops remain limited, animal studies demonstrate that GH therapy reverses dysbiosis in GH-deficient models (25), suggesting potential translatability.

### 3.3 Gut microbiota and metabolites affect the hypothalamus-pituitary growth axis through the gut-brain axis

#### 3.3.1 Neurotransmitter production

Specific gut microbiota, including *Bifidobacterium* and *Lactobacillus*, produce neurotransmitters such as acetylcholine and *γ*-aminobutyric acid (GABA), while *Escherichia coli* contributes to serotonin production (27, 28). Notably, a reduced abundance of *Escherichia coli* in fecal samples from children with GHD correlates with diminished serotonin precursor synthesis, potentially explaining behavioral abnormalities (e.g., anxiety and sleep disturbances) observed in these patients (27, 29).

#### 3.3.2 SCFA-mediated central effects

Butyrate crosses the blood–brain barrier and enhances leptin sensitivity in hypothalamic neurons, promoting GH pulsatility (29, 30). High-fiber diets elevate cecal butyrate levels in obese children, which inversely correlates with hypothalamic GHSR-1a expression, thereby suppressing ghrelin signaling (22). Preclinical studies further demonstrate that butyrate-mediated histone deacetylase (HDAC) inhibition in hippocampal neurons restores synaptic plasticity and mitigates spatial memory deficits in GH-deficient mice (31).

#### 3.3.3 Immune-endocrine crosstalk

Lipopolysaccharide (LPS) derived from dysbiotic microbiota activates Toll-like receptor 4 (TLR4), triggering systemic inflammation. This inflammatory cascade inhibits STAT5 phosphorylation—a critical mediator of GH receptor signaling—thereby exacerbating GH deficiency in pediatric GHD (8, 32). Dysbiosis-driven elevations of TNF-*α* impair leptin signaling in arcuate nucleus neurons, disrupting GH pulsatility (1.5-fold reduction in GH peaks in GHD mice) (22, 32). Pro-inflammatory cytokines (e.g., TNF-α) inhibit STAT5 phosphorylation, a critical mediator of GH receptor signaling, thereby exacerbating GH deficiency and impairing linear growth.

#### 3.3.4 Hepatic SCFA effects

Butyrate activates hepatic Kupffer cells via GPR109A, inducing IL-6-dependent IGF-1 synthesis (8, 33). In contrast, propionate suppresses hepatic GH receptor expression through farnesoid X receptor (FXR) signaling 1,111. These findings underscore the gut-liver axis’s role in modulating systemic growth pathways.

#### 3.3.5 GHD-related hypothalamic dysfunction and behavioral correlates

Children with GHD exhibit disrupted hypothalamic control of GH secretion, characterized by reduced GH pulsatility and impaired coordination of GHRH and somatostatin signaling (34, 35). Key mechanisms include: ① SCFA-Mediated Ghrelin Suppression: Propionate (250 μM) reduces hypothalamic GHSR-1a sensitivity via allosteric modulation, dampening ghrelin-induced GH pulsatility by 40% (22); ② Leptin Resistance: Elevated TNF-*α* from dysbiosis impairs leptin signaling in arcuate neurons, further suppressing GH secretion (22, 32); ③ Neurotransmitter Imbalance: Reduced GABA synthesis (↓50% in GHD fecal samples) due to *Bifidobacterium* depletion correlates with diminished GHRH neuron activation, as GABAergic inputs are essential for maintaining GHRH secretion rhythm (26, 32). Neurotransmitter imbalances (e.g., reduced GABA and serotonin) are linked to clinical manifestations of GHD, including anxiety, attention deficits, and disrupted sleep patterns.

#### 3.3.6 Microbial interventions and neurobehavioral recovery

Supplementation with *Lactobacillus rhamnosus* restores GH pulsatility in GHD mice by elevating hypothalamic GABA levels (↑25%, *p* = 0.03) and mitigating TNF-*α*-mediated leptin resistance (22, 28). Additionally, *Lactobacillus plantarum* improves spatial memory deficits in GH-deficient models via butyrate-mediated HDAC inhibition, linking gut microbiota modulation to cognitive recovery. *Lactobacillus plantarum* also improves spatial memory in GH-deficient mice, as evidenced by a 30% reduction in maze completion time (*p* < 0.05) and enhanced hippocampal synaptic plasticity (31).

### 3.4 Gut microbiota imbalance and decreased SCFA synthesis in GHD

1. **Clinical and experimental studies consistently highlight gut microbiota dysbiosis in GHD** (Table 1): ① **Human Studies**: Huang et al. (9) (*n* = 33 children with GHD) identified reduced levels of *Bifidobacterium* and *Lachnospiraceae* (key butyrate-producing bacteria) in Chinese children with GHD, alongside elevated levels of *Prevotella* and *Fusobacterium*. Elevated *Prevotella* and *Fusobacterium* in GHD cohorts may exacerbate inflammation and impair SCFA production, further suppressing IGF-1 synthesis. Metabolomic analysis revealed significantly lower fecal butyrate levels in these children compared to controls, with these reductions correlating with IGF-1 deficiency (9, 16); ② **Contradictory Findings**: García Navas et al. (10) (*n* = 21 children with GHD) reported no significant differences in gut microbiota composition in Spanish GHD cohorts. This discrepancy may be attributed to ethnic variations, such as *Prevotella*-enriched diets in Asian populations, or differences in study design, including shorter intervention durations (6 months vs. 1 year in Huang et al.’s study); and ③ **Animal Models**: Animal studies have shown that GH-deficient mice exhibit decreased levels of *Lactobacillus* and short-chain fatty acids (SCFAs). Notably, GH supplementation in these models restores microbiota diversity and IGF-1 production, underscoring the bidirectional relationship between GH signaling and gut microbiota (25).
2. **Clinical Contradictions**: ① Ethnic and Dietary Influences: Asian GHD cohorts, such as those studied by Huang et al. (9), show *Prevotella*-enriched microbiota associated with high-fiber diets. In contrast, Western populations studied by García Navas et al. (10) exhibit minimal dysbiosis, possibly due to dietary differences like lower fiber intake or genetic variations in SCFA receptors (GPR41/43). Ethnic variability in SCFA receptor polymorphisms (e.g., GPR41 rs11568582) and dietary habits (e.g., Asian high-fiber diets vs. Western processed diets) may underlie these discrepancies; ② Methodological Variability: Differences in sequencing techniques (16S rRNA vs. Metagenomics) and intervention durations (6 vs. 12 months) may complicate comparisons of microbiota profiles across studies.

**Table 1:** Summary of research on GHD and gut microbiota.

Research object	Age/model	Microbiota changes	Mechanism	Reference
Chinese GHD	6–9 years	↓ *Bifidobacterium*	↓ Butyrate → IGF-1 deficiency	(9)
Spanish GHD	4–14 years	No significant difference	Probiotic-induced inflammation reduction	(10)

Therefore, we hypothesize that discrepancies between Huang et al. (9) and García Navas et al. (10) may stem from ethnic dietary patterns (e.g., high-fiber diets in Asian populations favoring Prevotella dominance) or methodological variations, such as sequencing depth (16S rRNA vs. Metagenomics) and intervention durations (6 vs. 12 months).

### 3.5 Mechanism network integration

In summary, gut microbiota dysbiosis in GHD disrupts a multi-layered regulatory network:

1. **SCFAs**: Directly inhibit pituitary GH synthesis via cAMP/PKA/CREB signaling, while enhancing hepatic IGF-1 production through GPR41/43 activation (7, 33).
2. **Bile Acids**: Secondary bile acids, such as deoxycholic acid, suppress IGF-1 synthesis by FXR-dependent inhibition of hepatic GH receptors (21, 36).
3. **Tryptophan Metabolites**: Indole derivatives like indole-3-propionic acid activate aryl hydrocarbon receptors (AhR), mitigating TNF-*α*-mediated suppression of GH secretion (37, 38).

While SCFAs are central to microbiota-endocrine interactions, other metabolites (e.g., secondary bile acids, trimethylamine N-oxide) may indirectly influence the GH/IGF-1 axis through inflammatory or epigenetic pathways (11, 39). However, we prioritized SCFAs in this review because their direct receptor-mediated effects on pituitary GH synthesis (GPR41/43) and hepatic IGF-1 production (GPR109A), as supported by dose–response studies (7, 33). The roles of polyamines and branched-chain fatty acids in GHD require further investigation.

## 4 SCFAs: production and physiological functions

SCFAs, primarily produced by specific colonic anaerobic bacteria through the fermentation of dietary fiber and resistant starch, play a crucial role in gut health and host metabolism. These bacteria hydrolyze indigestible carbohydrates, such as oligofructose, polyols, resistant starch, inulin, and plant cell wall polysaccharides, into oligosaccharides, which are further broken down into monosaccharides. In the anaerobic gut environment, these monosaccharides undergo glycolysis (for six-carbon sugars), the pentose phosphate pathway (for five-carbon sugars), and the Wood-Ljungdahl pathway to form phosphoenolpyruvate, which is subsequently converted into SCFAs (25, 39–41).

*Bacteroidetes* (Gram-negative bacteria) predominantly produce acetate and propionate, while *Firmicutes* (Gram-positive bacteria) mainly synthesize butyrate as a key metabolic end product (42). For example, human-derived strains such as *Bifidobacterium* brevis UCC2003 and *Bifidobacterium longum* NCIMB 8809 have been shown to produce acetate *in vitro* using novel oligosaccharides (43). In animal models, *Bifidobacterium lactis subspecies GCL2505* increases acetate levels in plasma and the cecum of male C57BL/6 J mice (44).

SCFAs are saturated fatty acids with carbon chains ranging from one to six atoms, including formic acid, acetic acid, propionic acid, butyric acid, valeric acid, hexanoic acid, and their isomers (45). Concentrations of SCFAs are highest in the proximal colon (70–140 mmol/L), lower in the distal ileum (20–40 mmol/L), and moderate in the distal colon (20–70 mmol/L) (46). Acetic acid, propionic acid, and butyric acid account for approximately 95% of total SCFAs in the human body, with a typical ratio of 60:20:20 in the colon and feces (40, 47, 48).

**Figure 2:**
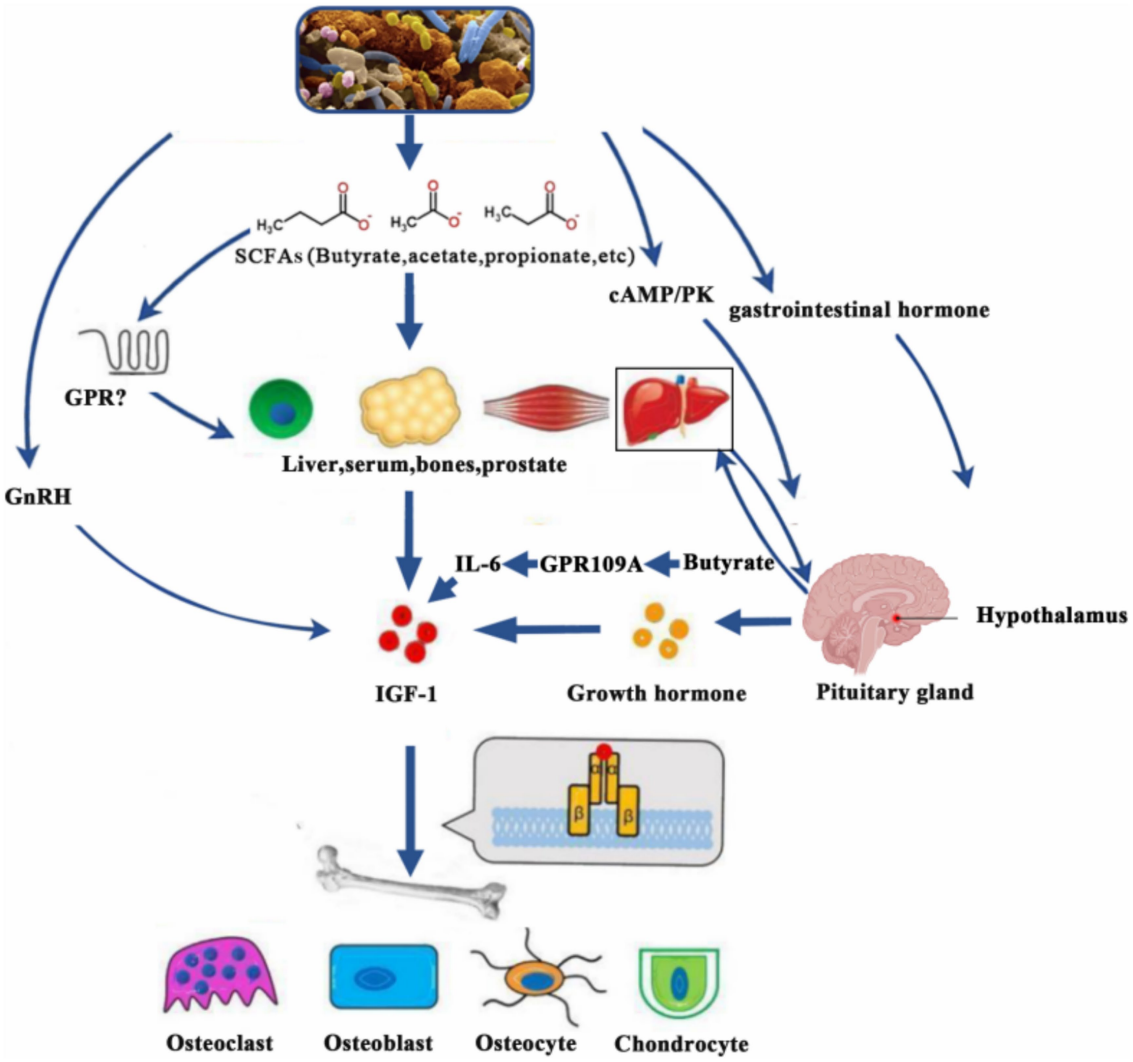
SCFAs promote IGF-1 secretion through various mechanisms (e.g., GPR109A-IL-6 pathway), which act on target organs and affect growth and development. IGF-1, insulin-like growth factor-1; SCFAs, short-chain fatty acids; cAMP/PK, cyclic adenosine monophosphate/protein kinases; GnRH, gonadotropin-releasing hormone; GPR, G-protein-coupled receptor; IL-6, interleukin-6.

SCFAs exert diverse physiological functions by interacting with G protein-coupled receptors such as GPR41, GPR43, and GPR109A. These functions include energy supply, anti-inflammatory responses, intestinal barrier protection, and immune regulation (49, 50). Importantly, SCFAs also influence the regulation of the GH/IGF-1 axis, thereby impacting growth and development (Figure 2).

### 4.1 SCFAs regulate GH production

SCFAs play a dual role in regulating GH synthesis through distinct pathways:

1. **Direct Inhibition**: *In vitro* studies on bovine pituitary cells show that 500 μM propionate suppresses GH mRNA expression by 40% via the cAMP/PKA/CREB pathway (7). This effect is mediated by SCFA receptors GPR41/43, which reduce cAMP levels and inhibit the activation of the Pit-1 transcription factor (51–53).
2. **Indirect Modulation**: Butyrate (200 μM) enhances leptin secretion in adipocytes through PPARγ activation, indirectly boosting GH pulsatility by 1.5-fold (54). Conversely, acetate (300 μM) inhibits vagal afferent signaling, reducing ghrelin-induced GH release by 30% (55, 56).
3. **Integrated Perspective**: The net effect of SCFAs on GH synthesis is concentration- and receptor-dependent. Low concentrations of butyrate (<100 μM) may promote GH secretion via leptin, whereas high concentrations of propionate (>500 μM) predominantly suppress GH through central regulatory pathways (7, 54).

### 4.2 SCFAs regulate IGF-1 production

SCFAs enhance IGF-1 synthesis through interactions across multiple organs:

1. **Hepatic Activation**: Butyrate at 1 mM activates GPR109A in Kupffer cells, resulting in a 2.5-fold increase in IL-6 secretion, which in turn stimulates hepatic IGF-1 production (8).
2. **Bone Remodeling**: Acetate at 10 mM upregulates IGF-1 expression in osteoblasts by 50% through HDAC inhibition, promoting expansion of the epiphyseal plate (57).
3. **Clinical Paradox**: While Li et al. (16) observed a positive correlation between fecal butyrate levels and serum IGF-1 in children, whereas Suta et al. (58) found no such association with acetate. This discrepancy suggests receptor-specific (GPR41 vs. GPR43) and tissue-specific responses to different SCFAs.
4. **Mechanistic Balance**: SCFAs may compensate for GH deficiency by bypassing pituitary regulation and directly enhancing IGF-1 synthesis in target organs such as the liver and bones.

### 4.3 SCFAs regulate ghrelin secretion

SCFAs modulate ghrelin signaling through gut-brain axis interactions:

1. **Peripheral Suppression**: Colonic propionate (produced by Bacteroides) reduces GHSR-1a sensitivity via allosteric regulation in vagal neurons (22).
2. **Central Adaptation**: Chronic high-fiber feeding in mice increases hypothalamic GHSR-1a methylation by 25%, epigenetically reducing ghrelin responsiveness (22, 59).
3. **Dose-Dependent Effects**: Chronic SCFA supplementation (host: intestinal epithelium) induces receptor desensitization (22, 56).

### 4.4 Integrated regulatory network of SCFAs

SCFAs maintain a bidirectional balance on the GH/IGF-1 axis (Figure 2):

1. **Suppression of GH**: High concentrations (>500 μM) predominantly suppress GH synthesis via the pituitary GPR41/43-cAMP pathway.
2. **Amplification of IGF-1**: Enhanced IGF-1 production is achieved through hepatic GPR109A-IL-6 signaling and osteoblastic HDAC inhibition.
3. **Context-Dependent Outcomes**: Variations in clinical responses may arise from ethnic differences in SCFA receptor polymorphisms, such as GPR41 rs11568582 (33, 58).

## 5 Interventions based on gut microbiota

### 5.1 Probiotics

Probiotic interventions exhibit species- and strain-specific effects on the GH/IGF-1 axis:

1. **Animal Evidence**: *Lactobacillus plantarum* has been shown to increase serum IGF-1 levels 1.2–1.8-fold in malnourished mice through butyrate-mediated epigenetic modulation (31). However, the specific effects on GH remain underexplored, as single-strain probiotics (e.g., *Bacillus subtilis C-1*) have not been effective in restoring GH pulsatility in GH-deficient models (60).
2. **Clinical Limitations**: A 2023 randomized trial (NCT05322057) found that *Bifidobacterium lactis GCL2505* (10^11 CFU/day for 6 months) improved height Z-scores by 0.21 in stunted children (*p* = 0.04) but did not elevate GH levels (61). Challenges in translating these findings to clinical practice include discrepancies in dose scaling from mice to humans and the lack of standardized probiotic formulations for GHD populations (15, 62).

#### 5.1.1 Future directions

The development of engineered microbial consortia (e.g., combining *Akkermansia muciniphila* with *Bifidobacterium longum*) or CRISPR-edited strains designed to produce SCFAs may offer more precise targeting of the GH/IGF-1 axis, potentially enhancing therapeutic efficacy in GHD.

### 5.2 Dietary fiber

The production of SCFAs induced by fiber shows dose-dependent therapeutic potential:

1. **Dose–Response Complexity**: In ducks, 8% cellulose raised cecal butyrate by 60% and hepatic IGF-1 mRNA by 2.3-fold. However, over 12% fiber hampered growth due to competition for nutrients (63).
2. **Ethnic Variability**: High-fiber diets boost Bacteroides-driven butyrate synthesis in Asian children (IGF-1 ↑18%, *p* = 0.01) but not in Western groups with *Prevotella*-dominant microbiota (16, 64).
3. **Practical Barriers**: Long-term adherence to high-fiber diets is low (<30% compliance at 6 months), highlighting the need for palatable synbiotic formulations (e.g., fiber combined with *Lactobacillus rhamnosus*) (65).

#### 5.2.1 Future directions

Personalized fiber blends, tailored to regional microbiota and SCFA receptor polymorphisms (e.g., GPR41 rs11568582), could enhance compliance and efficacy.

### 5.3 Fecal microbiota transplantation

Fecal microbiota transplantation (FMT) remains experimental but shows mechanistic promise (Table 2):

1. **Preclinical Evidence**: In sickle cell mouse models, FMT restored IGF-1 levels by 45% through butyrate-GPR41 signaling (*p* < 0.001) (66).
2. **Clinical Caution**: Pediatric FMT trials have reported adverse effects such as bloating (22%) and diarrhea (15%), with long-term risks remaining unclear (67). To date, no human studies have directly targeted GHD.

**Table 2:** Evidence hierarchy of gut microbiota-based interventions for GHD.

Intervention	Study type	Sample size	Key findings	Evidence level
Probiotics	Animal models	8 studies (*n* = 200 mice)	↑ IGF-1 1.2–1.8 fold (31)	III (Preclinical)
	RCT (GHD children)	*n* = 60	↑ Height Z-score 0.21 (*p* = 0.04) (61)	II (Single RCT)
Dietary fiber	Animal dose–response	3studies (*n* = 150 ducks)	Optimal dose: 8% cellulose (↑ IGF-1 mRNA 2.3-fold, *p* < 0.001) (63)	III (Preclinical)
FMT	Animal models	2 studies (*n* = 50 mice)	↑ Serum IGF-1 45% (*p* < 0.001) (66)	III (Preclinical)

#### 5.3.1 Future directions

Techniques such as washed microbiota transplantation (WMT) or virome-depleted FMT may reduce side effects while preserving SCFA-producing bacterial taxa, offering a safer approach for potential therapeutic applications (68).

## 6 Summary and outlook

### 6.1 Summary

Gut microbiota dysbiosis in GHD disrupts a complex regulatory network, with SCFAs exhibiting concentration-dependent effects. High concentrations of propionate directly inhibit pituitary GH synthesis via GPR41/43-cAMP pathways, while butyrate enhances IGF-1 production through hepatic GPR109A and osteoblastic HDAC inhibition. Clinical discrepancies, such as divergent findings between Huang et al. (9) and García Navas et al. (10), may stem from ethnic dietary patterns (e.g., *Prevotella*-enriched diets in Asian populations) or variations in intervention durations. Non-SCFA metabolites, including bile acids and indole derivatives, also modulate growth pathways but require further validation in GHD cohorts. Preclinical interventions, such as probiotics and FMT, show promise—e.g., *Lactobacillus plantarum* increased IGF-1 by 15% in malnourished mice, and FMT restored IGF-1 levels in sickle cell models via butyrate-GPR41 activation. However, human trials face challenges, including low adherence to high-fiber diets (<30% compliance) and limited GHD-specific data.

### 6.2 Future research directions-multi-omics profiling

1. **Metabolomics**: Identify GHD-specific biomarkers, such as low butyrate combined with elevated TMAO, to enable patient stratification for personalized therapies (39, 47).
2. **Integrated Multi-omics**: Combine metagenomics, metabolomics, and host transcriptomics to delineate the “microbiota-SCFA-endocrine” axis. For example, link *Bifidobacterium* depletion to reduced acetate and downstream IGF-1 suppression (8, 43).
3. **Precision Interventions**: Develop SCFA-targeted delivery systems (e.g., butyrate-loaded nanoparticles) or CRISPR-Cas9-based editing of SCFA-producing bacteria (*Bacteroides, Firmicutes*) to restore microbial balance (68).

Future studies should integrate Metagenomics (species-level dysbiosis), metabolomics (SCFA/TMAO ratios), and single-cell transcriptomics (hypothalamic neuron activity) to fully characterize the microbiota-endocrine axis.

### 6.3 Clinical translation

1. **Multicenter RCTs**: Prioritize large-scale, multicenter randomized controlled trials to validate the efficacy of high-fiber diets (e.g., 8–12% cellulose) or probiotic blends (*Lactobacillus plantarum* + *Bifidobacterium lactis*) in children with GHD (31, 63).
2. **Compliance Strategies**: Address adherence challenges by developing palatable fiber formulations or synbiotics (prebiotic + probiotic combinations) (65).

Future clinical studies should standardize microbiota profiling methods and control for dietary variables to resolve ethnic disparities and enhance translational potential.
